# How lung infection leads to gut injury

**DOI:** 10.18632/oncotarget.6470

**Published:** 2015-12-04

**Authors:** Jian Wang, Zhigang Tian

**Keywords:** lung, influenza virus, infection, intestine, inflammation, Immunology and Microbiology Section, Immune response, Immunity

In 1979, John Bienenstock explored the origin of B cells in the mucosal tissues, including intestinal, respiratory and genital tract, and found that donor mice derived mesenteric lymph node B cells could be detected in most mucosal tissues of recipient mice, while peripheral lymph node B cells returned to their site of origin after adaptive transfer. Therefore, he presented a concept of “common mucosal immune system” and speculated that the mucosal immune system might be a system-wide “organ” in which the immune cells distributed throughout the body could interplay among different mucosal tissues [1]. The concept has been presented over 30 years, although some studies provided evidences to support it, there are still some questions need to be clarified. For example, what is the mechanism that regulates the immune cell migration among different mucosal tissues, and whether there are specific passages to connect different mucosal tissues and mediate immune cell migration [2]?

Influenza is a common infectious respiratory disease. The most common symptoms of influenza in clinic include cough, fever, headache, and weakness. Meanwhile, influenza often associates with gastroenteritis like symptoms, such as vomiting and diarrhea, in some influenza patients, especially in young children [3, 4]. It is well known that the respiratory and intestinal tracts are both mucosal tissues. Therefore, exploring the mechanism underlying these clinical manifestations in the intestine during a lung-tropic viral influenza infection may provide new evidences to support the concept of “common mucosal immune system” and uncover some puzzles involved in it.

In a mouse model of intranasal A/Puerto Rico/8/34 (PR8) influenza virus infection, we found that influenza virus infection caused immune injury in respiratory and intestinal mucosal tissues but not in non-mucosal liver and kidney tissues, which consistent with symptoms in influenza patients [5]. Furthermore, influenza virus could not be detected in the intestinal tract after intranasal infection, and infecting mice intragastrically with PR8 had no influence on the small intestine, which ruled out the possibility that influenza virus entered the intestinal tract and directly caused immune injury at this site. So, the mouse model of PR8 infection provides us a perfect platform to study the connection among different mucosal immune systems.

What causes intestinal immune injury in response to respiratory influenza virus infection? The imbalance of intestinal microbiota is often involved in the occurrence of intestinal inflammation [6]. In our study, the numbers of segmented filamentous bacteria and *lactobacillus/Lactococcus* were decreased after PR8 infection, while the numbers of *Enterobacteriaceae* were increased; Depletion of intestinal microbiota by antibiotic treatment could protect the small intestine and colon against influenza induced injury, indicating that respiratory influenza virus infection caused intestinal injury by altering the composition of the intestinal microbiota. Ichinohe et al. also reported that intestinal microbiota could promote immune response against influenza infection in the lung. Therefore, commensal bacteria could not be ignored in mucosal immunological research.

Considering that influenza v1rus infection specifically caused immune injury in the respiratory and intestinal mucosal tissues, but not in the non-mucosal liver or kidney tissues in our study, implying that an interconnected relationship existed between respiratory and intestinal tract. We found that the chemokine CCL25 specifically and highly expressed in the small intestine after influenza virus infection when compared with other tissues, including lung, liver and kidney; treating mice with anti-CCL25 neutralizing antibody could inhibit intestinal microbiota alternation and reduce intestinal injury during influenza virus infection; Moreover, the number of CCR9^+^CD4^+^ T cells simultaneously increased in the lung, mediastinal lymph node, small intestine, and colon after influenza virus infection. Therefore, CCL25/CCR9 axis mediated the recruitment of lung-derived CCR9^+^CD4^+^ T cells into the intestinal tract, which then altered the composition of intestinal microbiota and caused intestinal immune injury. However, the pathway by which lung-derived CCR9^+^CD4^+^ T cells migrated into the intestinal tract is still not clear, by blood vessel or by lymphatic vessel, which need further study.

In conclusion, the influenza-infected mouse model provides a good platform to study the mechanisms underlying how respiratory influenza virus infection causes gastroenteritis-like symptoms in human. Moreover, these observations provide further evidences to support the concept of “common mucosal immune system”.

Zhigang Tian: Institute of Immunology and The CAS Key Laboratory of Innate Immunity and Chronic Disease, School of Life Sciences and Medica I Center, University of Science & Technology of China, Hefei, Anhui, China; and Collaborative Innovation Center for Diagnosis and Treatment of Infectious Diseases, State Key Laboratory for Diagnosis and Treatment of Infectious Diseases, First Affiliated Hospital, College of Medicine, Zhejiang University, Hangzhou, Zhejiang, China
